# Deficiency of Thyroid Hormone Reduces Voltage-Gated Na^+^ Currents as Well as Expression of Na^+^/K^+^-ATPase in the Mouse Hippocampus

**DOI:** 10.3390/ijms23084133

**Published:** 2022-04-08

**Authors:** Sivaraj Mohana Sundaram, Romy Marx, Heiko M. Lesslich, Irmgard D. Dietzel

**Affiliations:** 1 Faculty of Chemistry and Biochemistry, Biochemistry II, Ruhr University, 44780 Bochum, Germany; sivaraj.mohanasundaram@ruhr-uni-bochum.de (S.M.S.); romy_marx@bmas.bund.de (R.M.); heiko.lesslich@ruhr-uni-bochum.de (H.M.L.); 2 International Graduate School for Neuroscience, Ruhr University, 44780 Bochum, Germany; 3 Nanoscopy Group, Central Unit for Ionbeams and Radionuclides (RUBION), Ruhr University, 44780 Bochum, Germany

**Keywords:** thyroid hormone, congenital hypothyroidism, hippocampal neurons, Na^+^ current density, Na^+^/K^+^-ATPase, mouse brain, triiodo-L-thyronine

## Abstract

Mice lacking functional thyroid follicular cells, *Pax8^−/−^* mice, die early postnatally, making them suitable models for extreme hypothyroidism. We have previously obtained evidence in postnatal rat neurons, that a down-regulation of Na^+^-current density could explain the reduced excitability of the nervous system in hypothyroidism. If such a mechanism underlies the development of coma and death in severe hypothyroidism, *Pax8^−/−^* mice should show deficits in the expression of Na^+^ currents and potentially also in the expression of Na^+^/K^+^-ATPases, which are necessary to maintain low intracellular Na^+^ levels. We thus compared Na^+^ current densities in postnatal mice using the patch-clamp technique in the whole-cell configuration as well as the expression of three alpha and two beta-subunits of the Na^+^/K^+^-ATPase in wild type versus *Pax8^−/−^* mice. Whereas the Na^+^ current density in hippocampal neurons from wild type mice was upregulated within the first postnatal week, the Na^+^ current density remained at a very low level in hippocampal neurons from *Pax8^−/−^* mice. *Pax8^−/−^* mice also showed significantly decreased protein expression levels of the catalytic α1 and α3 subunits of the Na^+^/K^+^-ATPase as well as decreased levels of the β2 isoform, with no changes in the α2 and β1 subunits.

## 1. Introduction

The most severe form of hypothyroidism, congenital hypothyroidism, occurs in about every 3000th birth mainly due to thyroid dysgenesis or even thyroid agenesis [1,2,3,4]. The vast majority of cases of congenital hypothyroidism (CH) occur sporadically in children born to euthyroid mothers [1,2,5,6]. The transcription factors: thyroid transcription factors 1 and 2 (TTF1/TTF2) and paired box gene 8 (*PAX8*) play critical roles in thyroid development, according to recent studies [5,6,7,8]. In the absence of transcription factor TTF1, the thyroid gland, including the C-cells that produce calcitonin, will not form, while the absence of transcription factor *PAX8* will result in thyroid agenesis, resulting in athyreotic *Pax8^−/−^* pups dying within a few weeks of birth [5,6,7,9,10]. Humans carrying heterozygous mutations in the *PAX8* gene are at larger risk of developing thyroid malformations [9,11,12]. However, mice heterozygous for *Pax8* (*Pax8^+/−^*) do not manifest these malformations. Despite this interspecies difference, *Pax8^−/−^* mice are lethally hypothyroid. They show a delayed weight growth when compared to their *Pax8^+/−^* littermates and die within 25 days after birth [9,13,14,15,16]. Following treatment with T4 (thyroxine), *Pax8^−/−^* pups are able to survive beyond the weaning period, providing evidence that the *Pax8* deletion specifically impacts thyroid development [9,10]. Considering that the *Pax8^−/−^* mouse does not have thyroid hormone-producing follicular cells, it acts as a suitable animal model to study the consequences of congenital hypothyroidism.

In spite of the fact that hypothyroidism can currently be prevented by controlled hormone substitution, the molecular mechanisms responsible for the characteristic mental slowdown remain elusive [17,18,19,20,21,22]. Clinical electroencephalographic (EEG) data indicate that hypothyroid patients display a slowing of the α-rhythm, evoked potentials, and peripheral conduction velocity [23,24,25], as well as a characteristic slowing of the speed of speech [26]. As result of the central hypothyroid status, both peripheral and cortical excitability are reduced simultaneously [27,28,29,30,31]. Since hypothyroidism is characterized by slowed neuronal function, we previously investigated whether thyroid hormone regulates neuronal Na^+^ current density, which is the main determinant of the action potential upstroke velocity and also determines neuronal conduction velocity. We found that Triiodo-L-thyronine (T3) upregulates Na^+^ current density in cultured neurons from postnatal rats, while voltage-activated K^+^ currents were unchanged [32]. This is in line with investigations in human fetal neuroepithelial cells, which also found that T3 acts as a Na^+^ current regulator [33], and with observations from skeletal and heart cells [34,35,36]. In addition, we confirmed that Na^+^ currents from cortical neurons are regulated in the same manner and that the Na^+^ current density is upregulated in acutely dissociated neurons from hyperthyroid pups, whereas it is downregulated in cortical neurons isolated from hypothyroid pups [37], suggesting that the Na^+^ current regulation also occurs in vivo. Consistently, in hippocampal slices from thyroid hormone-deficient rats, decreased action potential upstroke velocities have been observed [38], in addition to further A-type potassium and calcium current regulations [39]. Hence, the finding that action potentials from neurons grown in the presence of T3 display increased upstroke velocity and amplitude, as well as higher discharge frequencies and vice versa [37] could potentially explain the decreased cortical excitability that has been measured in hypothyroid humans using transcranial magnetic stimulation [30]. It is conceivable that a dramatic reduction of ion channels could eventually lead to coma and death, which can occur in extreme cases of hypothyroidism [40].

One of the most well-known effects of thyroid hormone is its effect on oxygen consumption, roughly 40% of which is consumed to fuel the Na^+^/K^+^-ATPases [41,42,43,44,45,46,47,48]. Investigations of Ismail-Beigi and Edelmann failed to find obvious effects of thyroid hormone on Na^+^/K^+^-ATPases in slices from adult rat brain [48]. However, hypothyroidism induced on postnatal day 1 in rats has been observed to cause significant reductions in Na^+^/K^+^-ATPase activity when measured in adult rats [49] and reduced the expression of Na^+^/K^+^-ATPases in rat synaptosomes within the first two postnatal weeks [50,51]. In the meantime, various antibodies against the different isoforms of this enzyme have become available [52,53,54,55,56,57,58], enabling more detailed investigations of their developmental expression [59].

Since the results compiled above were obtained from rats [32,37], the first aim of the present study was to test whether hypothyroidism affects Na^+^ current densities in mice as well. To account for potential effects of thyroid hormone on the Na^+^/K^+^-ATPases, we additionally used Western blots to analyze the protein expression of three α and two β subunits of Na^+^/K^+^-ATPases. Our experiments were performed in hippocampal tissue from *Pax8^−/−^* mice serving as a suitable animal model for extreme hypothyroidism. Asymptomatic *Pax8^+/−^* and *Pax8^+/+^* littermates served as controls.

## 2. Results

### 2.1. Effects of T3 on Na^+^ and K^+^ Current Densities

**Figure 1:**
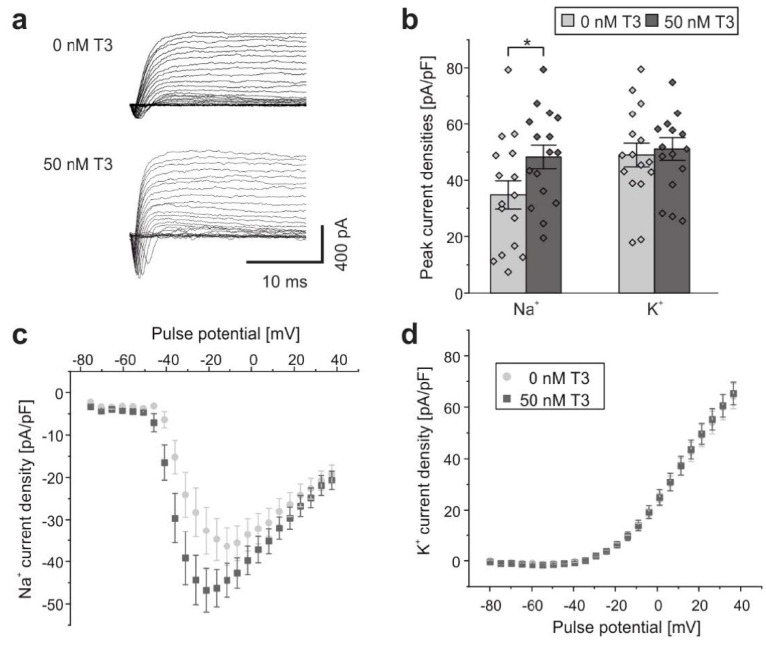
**Effects of T3 on Na^+^ and K^+^ currents in cultured mouse hippocampal neurons.** (**a**) Series of original Na^+^ and K^+^ current recordings from cultured *Pax8^+/+^*, *Pax8^+/−^* mouse hippocampal neurons evoked by step depolarizations in 5 mV increments starting from a holding potential of −80 mV (after liquid junction correction). Top: current traces from untreated neurons. Bottom: traces from neurons after treatment with 50 nM T3 for 4 days. (**b**) Peak Na^+^ and K^+^ currents normalized to membrane capacitance. Bars represent means ± SEM. Single data points shown as diamonds. Average current to voltage (I/V) relationships for (**c**) Na^+^ currents and (**d**) K^+^ currents normalized to membrane capacitance recorded from neurons cultured in presence or absence of 50 nM T3. Data points represent means ± SEM. Statistically significant differences marked by * (*p* < 0.05). Recordings from 16 neurons for each condition from two independent preparations.

The aim of the study was to investigate the impact of congenital hypothyroidism on currents through voltage-gated Na^+^ and potassium channels as well as expression of Na^+^/K^+^-ATPase isoforms in the hippocampus of *Pax8^−/−^* mice. This mouse model is effectively athyroid due to the absence of thyroid hormone-producing follicles [6]. In previous reports we had shown that in neonatal rat neurons from hippocampal neuron-glia mixed cultures, T3 leads to increased Na^+^ current densities compared with neurons cultured in absence of T3 [32,37]. However, so far, the effect of T3 on mouse hippocampal neurons remained to be validated. Therefore, as a first step, we tested whether the effect of T3 on cultured hippocampal neurons from mice yields similar results to that of previous investigations in rats. Neuron-enriched cultures, but still containing various types of glial cells, were obtained from P1–P6 old *Pax8^+/−^* and *Pax8^+/+^* mice. Na^+^ inward and K^+^ outward currents were recorded from untreated neurons and neurons that had been incubated with 50 nM T3 for 4 days (Figure 1a,b). Peak currents were measured at test potentials of −20 mV for Na^+^ and +20 mV for K^+^, and normalized to cell membrane capacitance yielding current densities (Figure 1b). While Na^+^ current densities of T3-treated hippocampal neurons were significantly increased by about 65% compared with control cultures (*p* < 0.05), K^+^ current densities remained unchanged when compared with currents recorded from untreated neurons (*p* > 0.715) (Figure 1d). Overall, the outcome of this part of the study demonstrated that treatment of cultured cells with T3 significantly increased Na^+^ current densities of *Pax8^+/−^* and *Pax8^+/+^* mice hippocampal neurons, whereas K^+^ current densities were not affected. These findings are in line with those shown in rats in our previous studies [32,37,60].

### 2.2. Na^+^ Current Densities in Hippocampal Neurons from Pax8^−/−^ Mice

**Figure 2:**
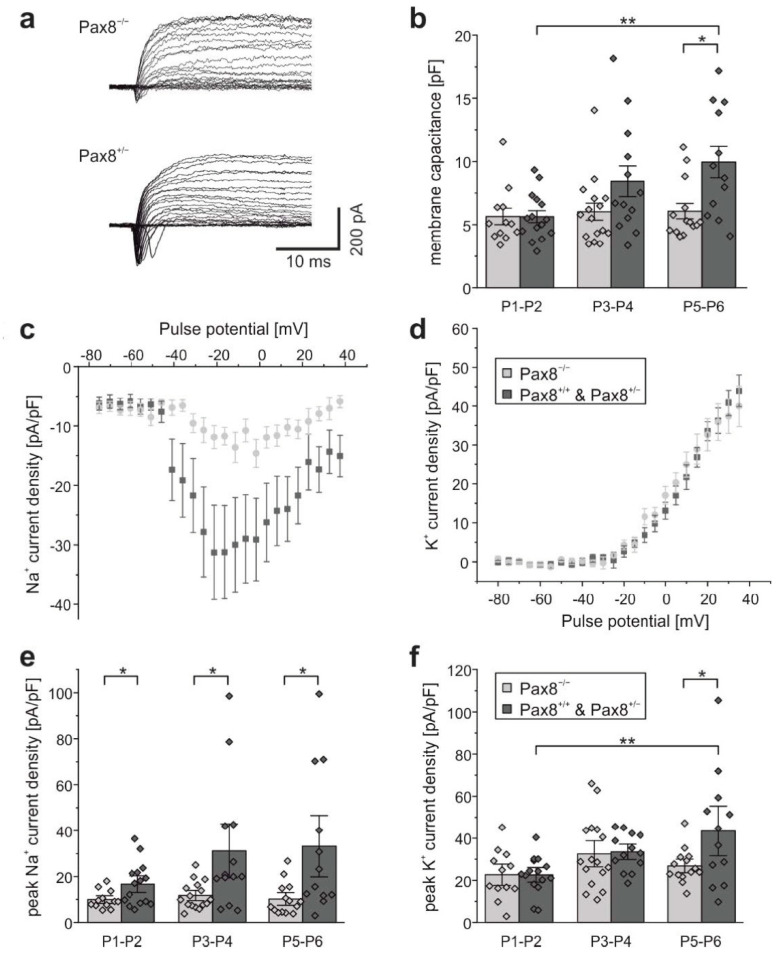
**Na^+^ and K^+^ currents in *Pax8^+/−^* and *Pax8^−/−^* mice.** (**a**) Exemplary series of original Na^+^ and K^+^ current recordings from acutely dissociated neurons elicited by step depolarizations in 5 mV increments starting from a holding potential of −80 mV (after liquid junction correction). Traces from P3–P4 hippocampal neurons obtained from a *Pax8^−/−^* mouse (top) and from a *Pax8^+/−^* mouse (bottom). (**b**) Membrane capacitances calculated from the integral of the charging curve for a test potential step of 20 mV. Bars represent means ± SEM; single data points shown as diamonds. Average current voltage (IV)-relationships for (**c**) Na^+^ and (**d**) K^+^ currents normalized to membrane capacitance recorded from acutely dissociated neurons from 3–4 day old *Pax8^+/+^* and *Pax8^+/−^* mice (dark gray), compared with currents from *Pax8^−/−^* mice (light gray). Data points represent means ± SEM. (**e**) Peak Na^+^ currents normalized to membrane capacitance recorded from acutely dissociated neurons at three postnatal time intervals. (**f**) Peak K^+^ currents normalized to membrane capacitance. Statistically significant differences marked with * (*p* < 0.05) and ** (*p* < 0.01). Bars represent means ± SEM. Data from individual neurons depicted as diamonds. Recordings from 12–16 neurons from at least three independent preparations of P1–P2, P3–P4, and P5–P6 mice. *Pax8^+/+^* and *Pax8*^+/−^ mice (dark gray); *Pax8^−/−^* mice (light gray).

Next, we studied whether the lack of thyroid hormone-producing follicles in newborn *Pax8^−/−^* mice might influence expression of Na^+^ and K^+^ channels in vivo. For this purpose, we measured Na^+^ and K^+^ currents from acutely dissociated hippocampal neurons of mice at ages P1 to P6 using the whole cell configuration of the patch-clamp technique. We further subdivided the data into the groups P1–P2, P3–P4, and P5–P6 and compared Na^+^ and K^+^ current densities of the *Pax8^−/−^* genotype with those of the control group consisting of *Pax8^+/−^* and *Pax8^+/+^* mice (Figure 2).

Na^+^ current densities were larger in hippocampal neurons from control mice compared to neurons from *Pax8^−/−^* mice at P1–P2 (*Pax8^−/−^*: 9.96 ± 1.13 pA/pF, *n* = 12; control: 16.70 ± 2.41 pA/pF, *n* = 15), P3–P4 (*Pax8^−/−^*: 11.74 ± 1.47 pA/pF, *n* = 16; control: 31.21 ± 7.84 pA/pF, *n* = 13) and P5–P6 (*Pax8^−/−^*: 10.24 ± 6.98 pA/pF, *n* = 14; control: 33.24 ± 8.91 pA/pF, *n* = 12). In all age categories, the differences in Na^+^ current densities were significant (P1–P2: *p* = 0.028; P3–P4: *p* = 0.012; P5–P6: *p* = 0.012) (Figure 2e). Interestingly, Na^+^ current densities in neurons from *Pax8^−/−^* mice remained constant and did not further increase with age, whereas in control mice, Na^+^ current densities were insignificantly (*p* = 0.059) larger at P3–P4 and P5–P6 compared with P1–P2 (Figure 2e). 

### 2.3. K^+^ Current Densities in Hippocampal Neurons from Pax8^−/−^ Mice

In contrast to Na^+^ current densities, no significant differences in K^+^ current densities between *Pax8^−/−^* mice and control mice were observed in P1–P2 and P3–P4 (Figure 2f). However, in P5–P6 mice, K^+^ current densities were significantly larger in control neurons compared to neurons from *Pax8^−/−^* mice (*p* < 0.026). K^+^ current densities increased slightly with the age of the pups, although these changes were not statistically significant until reaching P5–P6 (*p* < 0.0092). These results further support previous findings that voltage-gated Na^+^ channel expression of hippocampal neurons is highly dependent on the availability of thyroid hormone. On the contrary, the current density of delayed rectifier K^+^ channels was not significantly influenced by thyroid hormone during the first 4 days after birth (Figure 2f). 

### 2.4. Membrane Capacitance in Hippocampal Neurons from Pax8^−/−^ Mice

In Figure 2b, membrane capacitances of acutely dissociated hippocampal neurons from *Pax8^−/−^* mice and control animals are shown. At P1–P2, capacitances of neurons from both *Pax8^−/−^* and control pups were equally large. Moreover, membrane capacitances were found to increase with the age of the pups and were found to be significantly larger in P5–P6 mouse neurons compared to P1–P2 (*p* < 0.0017). While this was the case for neurons from control mice, capacitances in neurons from *Pax8^−/−^* mice did not seem to change with age. At P5–P6, membrane capacitances differed significantly between knockout and control mice (*p* < 0.05) (Figure 2b). This suggests that the size of neuronal cell bodies did not increase in cells from *Pax8^−/−^* mice. Although the cell bodies had been freshly dissected and thus had been deprived of most of their processes, these results are in line with morphological studies showing reduced cell body volumes of hippocampal neurons in postnatal hippocampus from rat [61].

### 2.5. Expression of Na^+^/K^+^-ATPase Isoforms in Hippocampal Tissue from Pax8^−/−^ Mice

**Figure 3:**
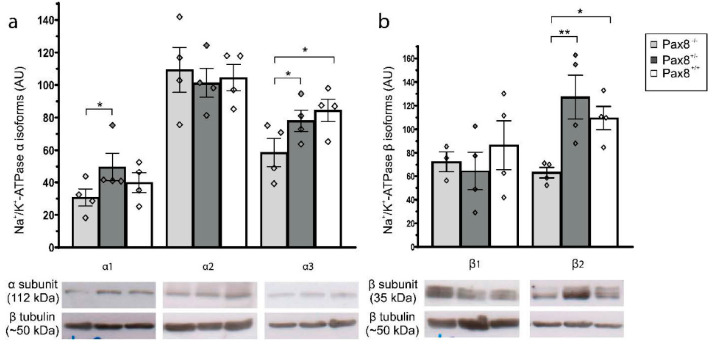
**Effects of congenital hypothyroidism on sodium potassium ATPase α and β subunit expression.** Protein levels of Na^+^/K^+^-ATPases (**a**) α1, α2, and α3 isoforms, and (**b**) β1 and β2 isoforms from P19–P21 old *Pax8^−/−^*(light gray), *Pax8^+/−^* (dark gray), and *Pax8^+/+^*(white) mice. Exemplary immunoblotting bands are shown below bar charts. β tubulin was used as internal control. Bars represent means ± SEM. Data from individual animals depicted as diamonds. Statistically significant differences marked with * (*p* < 0.05) or ** (*p* < 0.01), *n* = 3–4 for each condition.

Na^+^/K^+^-ATPase plays an important role in restoring the intracellular concentrations of both Na^+^ and K^+^ after neuronal excitation. Since acutely dissociated hippocampal neurons from *Pax8^−/−^* mice exhibited significantly smaller Na^+^ currents compared with wild-type and *Pax8^+/−^* mice, we investigated whether Na^+^/K^+^-ATPase expression is also influenced by congenital hypothyroidism. Since smaller Na^+^ currents translate into smaller intracellular Na^+^ concentration changes, less Na^+^/K^+^-ATPase might be necessary to maintain a low intracellular Na^+^ level. Furthermore, it has been previously found that thyroid hormone differentially regulates Na^+^/K^+^-ATPase α and β isoforms on mRNA as well as protein expression levels in different rat brain regions [62,63,64], and in cultured neurons from fetal rat brain [65]. However, expression of Na^+^/K^+^-ATPase isoforms in congenitally hypothyroid animal models has not been tested in detail. Therefore, we analyzed the protein expression profiles of Na^+^/K^+^-ATPase α and β isoforms in hippocampus from *Pax8^−/−^*, *Pax8*^+/−^, and *Pax8^+/+^* mice by immunoblotting (Figure 3). Since expression levels of Na^+^/K^+^-ATPases are much higher in adult than in neonatal brain tissue [59], these experiments were performed in animals as old as possible to obtain clear effects. Relative expression levels of Na^+^/K^+^-ATPase isoforms α1, α2, α3, β1, and β2 were quantified in total cell lysates from hippocampi obtained from P17 to P20 old *Pax8^−/−^*, *Pax8^+/−^*, as well as *Pax8^+/+^* mice. Figure 3a depicts immunoblots and corresponding mean values of protein expression levels of Na^+^/K^+^-ATPase α isoforms. The expression levels of Na^+^/K^+^-ATPase α1 were significantly reduced in *Pax8^−/−^* mouse hippocampi compared with *Pax8^+/−^* mice (α1: *Pax8^+/−^* vs. *Pax8^−/−^*, *p* < 0.0496; *Pax8^+/+^* vs. *Pax8^−/−^*, *p* < 0.4917). Likewise, the expression levels of Na^+^/K^+^-ATPase α3 were significantly reduced in *Pax8^−/−^* mice compared with *Pax8^+/−^* as well as *Pax8^+/+^* mice (α3: *Pax8^+/−^* vs. *Pax8^−/−^*, *p* < 0.0286, *Pax8^+/+^* vs. *Pax8^−/−^*, *p* < 0.0129). In contrast, the Na^+^/K^+^-ATPase α2 isoform showed no changes when compared to *Pax8^+/−^* and *Pax8^+/+^* hippocampi (*Pax8^+/−^* vs. *Pax8^−/−^*, *p* > 0.999, *Pax8^+/+^* vs. *Pax8^−/−^*, *p* > 0.999). The expression of the β2 isoform (Figure 3b) was significantly reduced by more than 50% (*Pax8^+/−^* vs. *Pax8^−/−^*, *p* < 0.0075, *Pax8^+/+^* vs. *Pax8^−/−^*, *p* < 0.0424). In contrast, the Na^+^/K^+^-ATPase β1 isoform expression showed only insignificant changes (*Pax8^+/−^* vs. *Pax8^−/−^*, *p* = 0.2405, *Pax8^+/+^* vs. *Pax8^−/−^*, *p* > 0.999) (Figure 3b). The datasets presented in this article are included in the Supplementary Material (Figure S1, Tables S1–S3).

## 3. Discussion

Our present results showed that in hypothyroid cultures from postnatal mice, thyroid hormone up-regulates voltage-gated sodium currents in the same manner as in rats [32,37]. These experiments were performed in the presence of some glial cells, most prominently astrocytes in the cultures. Our previous experiments in postnatal cultures from rats had shown that factors secreted from astrocytes, most prominently FGF-2, but potentially additional growth factors, are necessary for the T3-induced up-regulation of Na^+^ current densities [60,66]. Na^+^ current regulation by thyroid hormone has been found in other excitable cells as well, but there seem to be differences compared with rodent brains. For instance, in zebrafish embryos, thyroxin regulates Na^+^ currents in embryogenesis and the blockage of these currents prevents further development of the embryo [67]. In these cells, however, the regulation occurs through T4-binding to integrin receptors in the membrane, activating phosphorylation cascades [68]. Likewise, in heart ventricle muscle, sodium currents are upregulated via membrane receptors in cats [36,69]. Although the common outcome is an upregulation of excitability by an up regulation of Na^+^ currents in excitable cells, regulatory cascades of different complexity seem to operate in neurons from rodents. It remains presently unclear whether an up-regulation of the expression of Na^+^ channels can account for the upregulation of the Na^+^ current density by thyroid hormone. In muscle cells, an enhanced [^3^H]-saxitoxin binding after exposure to thyroid hormone suggests that this is indeed the case [34,35]. Preliminary data from our own laboratory using quantitative PCR suggested that an upregulation of Nav1.6 might be involved in neurons [70].

After having confirmed that an incubation of the cultures for several days with T3 leads to an upregulation of the Na^+^ current density in hippocampal neurons from mice as well we now investigated whether a similar effect can be confirmed in the complex in vivo environment. To this aim we compared Na^+^ current densities from acutely isolated cells that had just attached to the culture dish for a long enough time to allow patch-clamp recordings. 

At first glance it became apparent that the capacitances measured after rupturing the membranes with the patch pipettes were significantly smaller in neurons from *Pax8^−/−^* mice compared with their wild-type siblings when investigated after P3. This indicates smaller cell membrane areas seen by the patch pipettes. In our previous investigations, carried out in cell culture, we only observed insignificant increases in membrane capacitance following a few days of thyroid hormone treatment [32,60], suggesting that a longer duration of severe hypothyroidism is required to induce morphological changes large enough to reflect in changes in capacitance. Morphological investigations indicated predominantly reductions in dendritic spines by hypothyroidism, as for instance observed in cortical neurons by Ruiz-Marcos et al. [71] and Berbel et al. [72]. Although Madeira et al. found no conspicuous changes in nuclear sizes in the granular layer in the hippocampus of hypothyroid rats [73], Rami et al. reported decreased soma sizes in hippocampal pyramidal cells [61], altogether suggesting that changes in cell capacitance of freshly dissociated cells might reflect a reduction in cytoplasmatic volume as well as neurite areas and number of soma near branches, which are mainly collected by the dissociation procedure.

Na^+^ current densities in acutely isolated cells were overall smaller than currents recorded from cultured cells. This could have resulted to some extent from the lack of recovery time after trypsinization, which could have resulted in the removal of some of the Na^+^ channels [74]. Currents from *Pax8^−/−^* mice were very small and in contrast to currents recorded from neurons of *Pax8^+/−^* or *Pax8^+/+^* animals did not increase within the first postnatal week (Figure 2). This is in contrast with the general finding of an increase in Na^+^ current density during postnatal development [75] and with time in culture [32]. In contrast, K^+^ currents from wildtype and knock-out mice did not show statistically significant differences during the first 4 postnatal days, but were significantly smaller in cells dissociated from P3–4 old *Pax8^−/−^* animals. Compared with our previous investigations on rats and the data shown in Figure 1, the differences between sodium current densities from *Pax8^−/−^* mice and control mice were even larger than the effects recorded after 4 days of thyroid hormone treatment in cultures. These findings suggest that in general, neurons from severely hypothyroid rodents show significantly reduced sodium current densities if investigated in culture or from freshly dissociated tissue. Since the down regulation of Na^+^ currents observed in the *Pax8^−/−^* knockout mice substantially exceeded the effects on Na^+^ currents observed in less severe cases of hypothyroidism and became even more prominent with an increasing age of the animals, we speculate that this regulation might substantially contribute to the lethality of this mutation.

One of the most prominent features of hypothyroidism is a down-regulation of the metabolic rate, which can substantially be accounted for by a decrease in Na^+^/K^+^-ATPases in several tissues but not the adult brain [48]. In contrast to adult rat brain, after hypothyroidism in the postnatal period, Valcana and Timiras had previously observed a decrease in Na^+^/K^+^-ATPase activity [49]. In synaptosomes from postnatal rats, Lindholm described an increase in Na^+^/K^+^-ATPases within the first 14 postnatal days by thyroid hormone [53].

In order to find out whether the expression of Na^+^/K^+^-ATPases is regulated in *Pax8^−/−^* mice as well, Western blots for the catalytic alpha-subunits as well as the β1 and β2 subunits were performed. We chose the oldest animals available, since we expected clearer results due to larger effects in older animals. Our results indicate that the ubiquitously expressed α1 subunit and the α3 subunit, which is specifically expressed in excitable cells, were down-regulated, whereas the α2 subunit, which is most prominently expressed in glial cells [76,77], was unaffected in *Pax8^−/−^* mice. This finding could reflect the decreased need for removal of Na^+^ from active neurons, while also indicating that the capacity to remove larger intracellular sodium loads from neurons (and to consume ATP) is impaired in brains of *Pax8^−/−^* animals. The largest effect observed, was however, a reduction of the β2-subunit in the hypothyroid *Pax8^−/−^* mice. This subunit has been suggested to be most prominently expressed together with α2-subunits, increase the cycle rate of the pump, and might be involved in potassium clearance by astrocytes around hyperactive neurons [78]. We might thus speculate that the decreased neuronal activity in hypothyroid mice might lead to an exchange in the subunit composition in astrocytes, which need less β2-subunits to remove extracellular K^+^. 

Evidence that the up-regulation of Na^+^/K^+^-ATPases could indeed be secondary to an upregulation of Na^+^ channels by thyroid hormone treatment was reported in muscle cells using [^3^H]-saxitoxin and [^3^H]-ouabain binding [79]. The activity of the Na^+^/K^+^-ATPases is the first step in establishing the membrane potential by maintaining the intracellular Na^+^ concentration at levels of approximately 10 mM and the intracellular K^+^ concentrations at about 100 mM [80,81]. It is activated by binding of 3 Na^+^ ions to its intracellular binding pockets if the intracellular Na^+^ activity increases due to Na^+^ influx through co-transporters or ligand- or voltage-gated ion channels. That changes in Na^+^ levels activate the pumps was first demonstrated by studies utilizing the Na-ionophore monensin that increases cellular Na^+^ and Na^+^/K^+^-ATPase activity [82,83,84,85]. An increased exposure to an enhanced Na^+^ load additionally stimulates the expression of increased numbers of Na^+^/K^+^-ATPases in the plasma membrane. Thus, evidence has been obtained that a rise in intracellular Na^+^ concentration using 50 µM of veratridine, which blocks the inactivation of voltage-gated sodium channels, upregulated the α3 Na^+^/K^+^-ATPase isoform in rat thalamic neurons [86]. It remains to be elaborated to which extent the reduced Na^+^ currents in hypothyroidism could account for the decreased expression of the α1, α3, and β2 subunits found in the present investigation. 

## 4. Materials and Methods

### 4.1. Mice

*Pax8^+/−^* mice were kindly provided by K. Bauer, Max Planck Institute for Experimental Endocrinology, Hannover, Germany and maintained as a breeding colony at the animal facility of the Department of Biochemistry II at the RUBION, Ruhr University, Bochum, Germany. All pups were kept with their mothers, which were housed under a 12 h light/dark cycle with free access to food and water. All animal procedures were in accordance with the German legislation. Recordings and tissue collections were performed between 2005–2013. The genotype at the *Pax8* locus was determined using 3 oligonucleotides (5′-GGA TGT GGA ATG TGT GCG AGG-3′, 5′-GCT AAG AGA AGG TGG ATG AGA G-3′, 5′-GAT GCT GCC AGT CTC GTA G-3′) and the following temperature schedule: 95 °C for 10 min (95 °C for 1.5 min, 61 °C for 2.5 min, 72 °C for 3 min, 35 times), 72 °C for 10 min. This amplifies a 390-bp fragment for the wild-type allele and a 370-bp fragment for the knockout allele.

### 4.2. Hippocampal Neuronal Culture

To prepare hippocampal cell cultures, hippocampi of P2–P3 wildtype, *Pax8^−/−^*, and *Pax8^+/−^* mice pups were removed under sterile conditions and collected in ice-cold modified phosphate buffered saline (MPBS) containing 137 mM NaCl, 2.7 mM KCl, 5 mM Na_2_HPO_4_, 0.89 mM KH_2_PO_4_, 10 mM HEPES, 1 mM pyruvate, 10 mM glucose, 1 mM L-alanyl-glutamine, and 1 mg/mL BSA, as well as 25 U/mL penicillin and 25 g/mL streptomycin (1% Penstrep). Additionally, 10 µg/mL deoxyribonuclease I and 5 µg/mL of a 2.5% Trypsin (Invitrogen) solution were added. After incubation of the tissue under gentle agitation for 7 min at 37 °C, cells were triturated 15 times using 1000 µL plastic pipette tips. The dissociated cells were collected in 10 mL MPBS and centrifuged at 145× *g* at room temperature for 12 min. The pellet was resuspended in RPMI-1640 medium (PAA, Cölbe, Germany) containing 10% fetal calf serum (FCS, Invitrogen), 1% Penstrep, 1% glutamine, and incubated at 37 °C with 5% CO_2_ in a humidified air chamber for 1 h. After pre-plating, the supernatant containing the neurons was collected in 10 mL RPMI-1640 containing 10% FCS, 1% Penstrep, and 1% glutamine, and centrifuged at 145× *g* at RT for 12 min. The pellet was resuspended in RPMI-1640 containing 10% FCS, 1% Penstrep, and 1% glutamine and plated on 3 cm diameter Petri dishes. After 1 day in vitro (DIV), the medium was replaced by fresh NB medium supplemented with 1% Penstrep, 2 mM L-alanyl-glutamine, and 2% of modified B18 (without T3) [87]. Cells were seeded on 3.5 cm diameter petri dishes coated with Poly-D-lysine (5 µg/mL in H_2_O for 1 h) at a density of 5–9 × 10^5^ cells/cm^2^ and incubated at 37 °C in a humidified air chamber (B5060 Incubator; Heraeus, Germany) with 5% CO_2_. Before preparation of cultures from individual animals, tail samples were collected for genotyping.

### 4.3. Cell Treatment

T3 (Sigma-Aldrich, Schnelldorf, Germany) was dissolved in 0.01 M NaOH. Cell culture medium was changed after 2 DIV, and cells were either incubated with 50 nM T3 or no T3 (control) for 4 days.

### 4.4. Acutely Dissociated Hippocampal Neurons

To obtain acutely dissociated neurons, hippocampi were removed from P1 to P6 old *Pax8^+/−^* and *Pax8^−/−^* mice and transferred to dissociation solution containing 130 mM NaCl, 1 mM CaCl_2_, 25 mM D-(+)-Glucose, 20 mM HEPES, 5 mM KCl, and 1 mM MgCl_2_. After cutting hippocampi into slices, not exceeding a width of 1 mm, 3% trypsin was added, and the slices were stirred mildly for 90 min at 32 °C in a water bath with additional carbogen supply. Subsequently, cells were equilibrated for 1 h with carbogen at 32 °C before slices were triturated in 1 mL dissociation solution saturated with carbogen and kept at RT for 1 h in a Petri dish coated with Poly-D-lysine prior to electrophysiological recordings.

### 4.5. Patch-Clamp Recording Conditions

Whole-cell recordings were performed with borosilicate pipettes (GB-150TF-8P; Science Products, Hofheim, Germany) pulled on a PP-830 puller (Narishige Europe, London, UK) yielding resistances between 4–5 MΩ after filling. Pipettes were filled with electrolytes composed of 100 mM K-gluconate, 1.1 mM EGTA, 0.1 mM CaCl_2_, 5 mM MgCl_2_, 5 mM NaCl,10 mM HEPES, and 3 mM Mg^2+^-ATP. Bath solutions contained 110 mM NaCl, 1.8 mM CaCl_2_, 10 mM Glucose, 10 mM HEPES, 5.4 mM KCl, and 0.8 mM MgCl_2_. Osmolarities of pipette and bath solutions were adjusted to the osmolarity of the NB medium. A liquid junction potential of this solution with respect to the bath solution of 15 mV was corrected offline. Peak Na^+^ currents were measured at a potential of −20 mV after correction of the liquid junction potential. Only Na^+^ currents showing an activation range exceeding 20 mV in the current to voltage relationship were included in the evaluation of the data to exclude recordings with very poor space clamp controls. K^+^ currents were determined as the mean current at the end of the test potential of +20 mV. Recordings were performed at RT using a L/M-EPC-7 amplifier (List Medical, Darmstadt, Germany). Series resistance errors were compensated by up to 30%. Signals were filtered using the EPC-7 10-kHz lowpass filter and then digitized at a sampling rate of 20 kHz. Current densities were determined by normalizing the respective current to the cell capacitance calculated from the integral of the charging curve for a test potential step of 20 mV after compensation of the electrode capacitance. Cells with series resistances above 20 MΩ were discarded to minimize series resistance errors. Leakage and capacitive artifacts were subtracted using a P/4 protocol. Data were digitized with a DigiData 1200 board (Axon Instruments, Union City, CA, USA) and stored on a personal computer. Data evaluation was performed with Clampfit 10.7.0.3 (Molecular Devices).

### 4.6. Hippocampal Tissue Preparation and Immunoblotting

P17-P20 *Pax8^+/−^* and *Pax8^−/−^* mice were sacrificed through cervical dislocation and hippocampi were collected and stored in −80° freezers. Tissue samples were lysed in buffer containing 1% NP-40, 0.1% Na^+^-Dodecyl sulphate, 0.5% Na^+^ deoxycholate, 150 mM NaCl, 2 mM EDTA, and 50 mM NaF supplemented with 10% protease inhibitor cocktail (Sigma, P2714-1BTL). Tissues were homogenized using an Ultra-Turrax homogenizer. Next, protein extracts were obtained by centrifugation of the tissue lysates at 12,000 rpm for 20 min at 4 °C. Protein concentration was measured using the Bio-Rad DCTM protein assay kit. Lysates were boiled with 1X Laemmli sample buffer containing 1.25% β-mercaptoethanol [88]. Afterwards, samples of 15 µg of protein were resolved by a 10% sodium dodecyl sulfate polyacrylamide gel electrophoresis and transferred to a nitrocellulose membrane [88]. Membranes were blocked in 5% dry milk powder in Tris-buffered saline (TBS) and incubated overnight at 4 °C with primary antibodies against Na^+^/K^+^-ATPase isoforms [anti-Na^+^/K^+^-ATPase α1, Millipore (05–369) dilution 1:2500; anti-Na^+^/K^+^-ATPases α2, Millipore (07–674) dilution 1:2500; anti-Na^+^/K^+^-ATPases α3, Millipore (06–172) dilution 1:2500; anti-Na^+^/K^+^-ATPases β1, Millipore (06–170) dilution1:2500; anti-Na^+^/K^+^-ATPases β2 Millipore (06–171) dilution 1:2500], and anti β-tubulin (Sigma/T4026, dilution 1:5000). After incubation with the primary antibodies, membranes were washed three times for 10 min in TBS containing 0.02% Tween-20 (TBS-T), followed by secondary antibody incubation with horseradish peroxidase-conjugated goat anti-rabbit (Cell signaling, 7074) and goat anti-mouse antibodies (Sigma, A9044) for 1 h at RT. Then, the membranes were washed with TBS-T three times and antibody detection was performed by using West Femto enhanced chemiluminescent solution (Thermo Scientific, Waltham, MA, USA). Blot intensities were quantified using Image J-1.46r (NIH, Bethesda, MD, USA). After background subtraction, protein expression of Na^+^/K^+^-ATPases α and β isoforms was normalized using β-tubulin as an internal control.

### 4.7. Statistical Analysis

Statistically significant differences between the data sets of the electrophysiological recordings (control versus T3-treated groups; control (*Pax8^+/+^* & *Pax8^+/−^*) versus *Pax8^−/−^* groups) were assessed by Student’s *t* test using GraphPad Prism version 8.0 for Windows (GraphPad Software, La Jolla, CA, USA). Electrophysiological data were collected from two individual hippocampal neuronal cultures or at least three acutely isolated hippocampal neuronal culture preparations. At least three mice per group were used for the immunoblotting part of this study. For the immunoblotting data sets, RM one-way ANOVA, followed by Bonferroni’s multiple comparisons test, was performed to calculate significant differences among the *Pax8^+/+^*, *Pax8^+/−^*, and *Pax8^−/−^* groups using GraphPad Prism version 8.0.

## 5. Conclusions

Taken together, our data suggest that the neuronal excitability and sodium pumping capacity are severely compromised in brains from mice lacking a thyroid gland, explaining the poor conditions of these animals. Since Na^+^/K^+^-ATPases are stimulated by the intracellular sodium load, which decreases with decreasing neuronal activity, this work sets the stage for further investigations of how currents through particular sodium channel alpha subunits could potentially lead to the upregulation of specific Na^+^/K^+^-ATPase isoforms.

## Data Availability

Excel files containing the data for Figure 1, Figure 2 and Figure 3 and original immunoblotting pictures are available online at https://www.mdpi.com/article/10.3390/ijms23084133/s1.

## Supplementary Materials

The following supporting information can be downloaded at: https://www.mdpi.com/article/10.3390/ijms23084133/s1.
