# Supplementary material for: Deficiency of Thyroid Hormone Reduces Voltage-Gated Na+ Currents as Well as Expression of Na+/K+-ATPase in the Mouse Hippocampus
Source: Int J Mol Sci. 2022 Apr 8;23(8):4133. doi: 10.3390/ijms23084133 (PMC9031557; doi:10.3390/ijms23084133)
Supplement: Supplementary file 1 [file ijms-23-04133-s001.zip › Sundaram et al 2022 - annotated blots_raw data_figure 3.pdf]

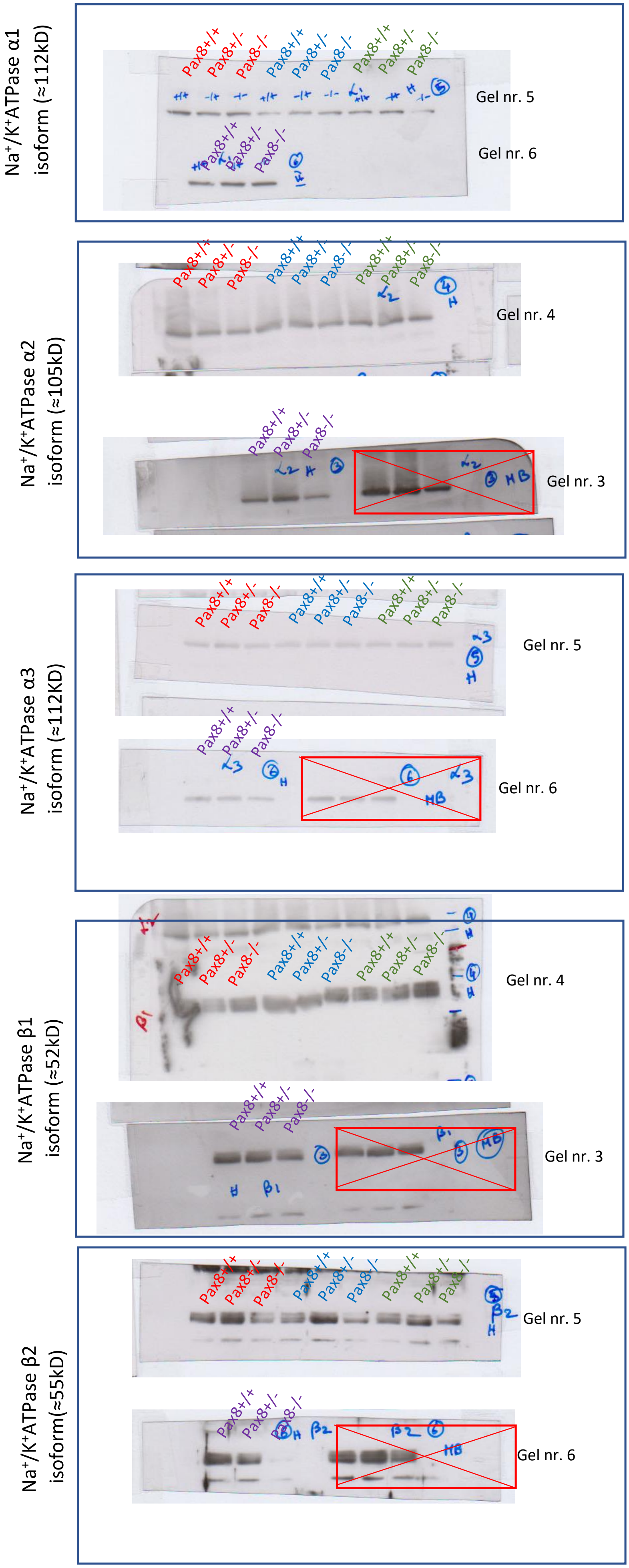

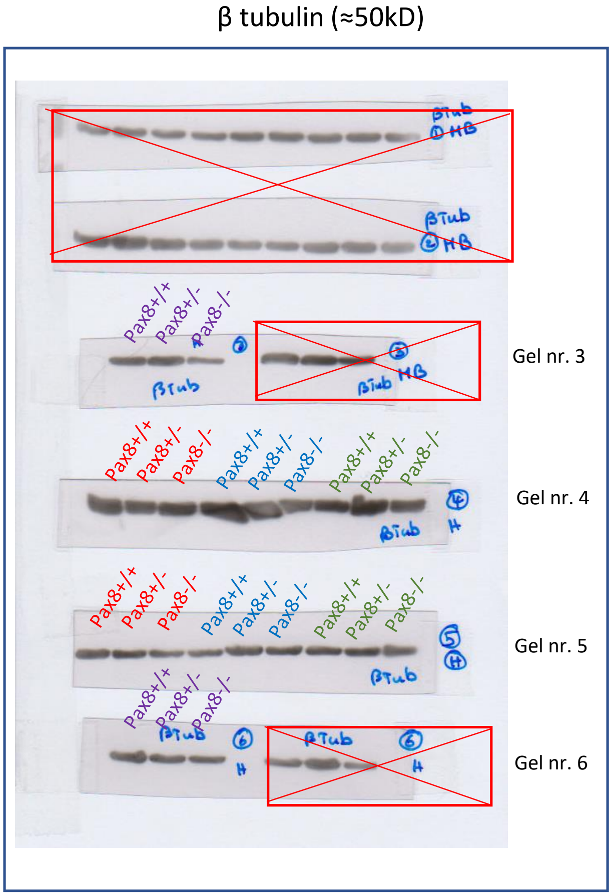

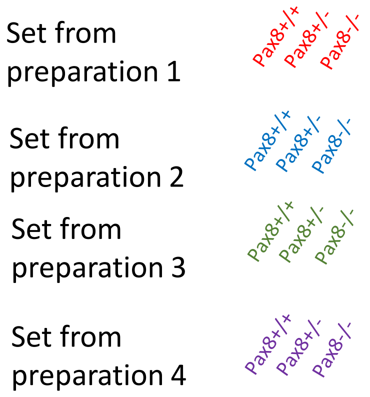

Note: crossed blots corresponding to β tubulin gels 1, 2, 3 or 6 were from midbrain tissues and thus not included in the present investigation on hippocampal tissues.
